# Attentional capture by task-irrelevant fearful faces is revealed by the N2pc component

**DOI:** 10.1016/j.biopsycho.2006.06.008

**Published:** 2007-01

**Authors:** Martin Eimer, Monika Kiss

**Affiliations:** School of Psychology, Birkbeck College, University of London, Malet Street, London WC1E 7HX, England, UK

**Keywords:** Attention, Event-related brain potentials, Emotion, Visual processing

## Abstract

We measured the N2pc component as an electrophysiological indicator of attentional selection to investigate whether fearful faces can attract attention even when they are entirely task-irrelevant and attention is focused on another demanding visual monitoring task. Participants had to detect infrequent luminance changes of the fixation cross, while ignoring stimulus arrays containing a face singleton (a fearful face among neutral faces, or neutral face among fearful faces) to the left or right of fixation. On trials without a target luminance change, an N2pc was elicited by fearful faces presented next to fixation, irrespective of whether they were singletons or not, demonstrating that irrelevant fearful faces can bias the spatial distribution of attention. The N2pc to fearful faces was attenuated when face arrays were presented simultaneously with a target luminance change, suggesting that concurrent target processing reduces attentional capture by emotional salient events.

It is often assumed that affectively salient stimuli have a specific ability to capture attention. Strong attentional biases towards emotional stimuli have indeed been found in behavioural studies investigating the attentional blink (e.g., Anderson and Phelps, 2001; Keil and Ihssen, 2004) or dot probe detection (e.g., Mogg and Bradley, 1999). Visual search experiments have provided further evidence for the capture of attention by fear-relevant stimuli. Angry faces presented among happy faces are detected faster than vice versa (Hansen and Hansen, 1988, but see Purcell et al., 1996). Similar results have also been obtained for schematic angry faces (Öhman et al., 2001b; see also Eastwood et al., 2001), and for fear-related non-face stimuli (snakes and spiders) presented among fear-irrelevant stimuli (Öhman et al., 2001a), suggesting that the emotional valence of a stimulus is processed outside the focus of attention and can guide attention to its location.

In previous visual search experiments investigating attentional capture by emotional stimuli, these stimuli were always task-relevant. The aim of the present study was to use event-related brain potential (ERP) measures to investigate the hypothesis that fear-related stimuli trigger attentional capture even when they can be completely ignored, and attention is actively engaged elsewhere. Stimulus arrays consisting of fearful and neutral faces were presented while participants were actively monitoring the fixation cross in order to detect a small change in its luminance that occurred on some trials simultaneously with the onset of the face array. Analogous to the visual search studies described above, face arrays contained either a single fearful among neutral faces, or a single neutral among fearful faces. These singletons were presented near fixation on the left or right side (see Fig. 1), but were entirely task-irrelevant. To detect any attentional capture triggered by irrelevant face singletons, we measured the N2pc component as an electrophysiological indicator of attentional selection. The N2pc is typically elicited at posterior electrodes between 180 and 300 ms after stimulus onset contralateral to the side of an attended visual event, such as a target in a visual search task (c.f., Luck and Hillyard, 1994; Eimer, 1996; Woodman and Luck, 1999), and is assumed to reflect spatially selective attentional processing.

We quantified the N2pc by measuring posterior ERPs contralateral and ipsilateral to the face singleton, separately for fearful and neutral singletons, and separately for trials with or without a target luminance change. If fearful faces were specifically able to attract attention even when they are task-irrelevant, an N2pc should be present in response to stimulus arrays containing a fearful face singleton. In contrast, no N2pc should be found for neutral singletons. If the degree of attentional capture by fearful faces was reduced on trials where a central target was presented simultaneously, the N2pc should be attenuated on luminance change trials. The absence of any N2pc to fearful face singletons would suggest that a narrow central attentional focus prevents attentional capture by irrelevant fear-related stimuli.

## 1 Methods

### 1.1 Participants

Nineteen volunteers took part in this experiment. Three were excluded because of eye movement artifacts or excessive alpha activity, leaving 16 participants (eight male, eight female, aged 20–41 years, mean age: 29 years) in the sample. All participants had normal or corrected visual acuity. Fifteen participants were right-handed, and one was left-handed.

### 1.2 Stimuli and procedure

**Fig. 1:**
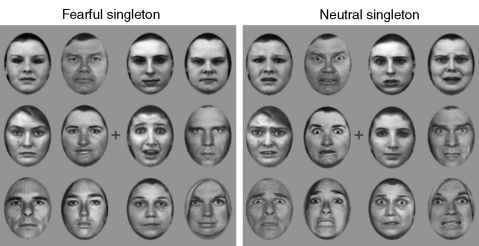
Examples of stimulus arrays: fearful singleton face among neutral faces (left panel); neutral singleton face among fearful faces (right panel).

Stimulus displays consisted of bilateral 3 × 4 arrays of greyscale faces (total size: 14° × 12.5°; size of each face: 2.6° × 3.6°) presented against a light grey background (28 cd/m^2^). Each stimulus array contained either a fearful singleton face among neutral faces (Fig. 1, left panel) or a neutral singleton face among fearful faces (Fig. 1, right panel) that was located next to the central fixation cross on the left or right side. Faces were drawn randomly without replacement from a set of 14 pictures of faces with neutral expression, and a set of the same 14 individuals with fearful expression (Ekman and Friesen, 1976). A central fixation cross (0.5° × 0.5° visual angle) was continuously present throughout the experimental blocks.

Twelve experimental blocks of 64 trials per block were run. On each trial, a face array was presented for 200 ms. The intertrial interval was 2000 ms. Participants were instructed to maintain fixation at all times, and to focus their attention on the fixation cross in order to detect an infrequent luminance change from dark grey (21 cd/m^2^) to light grey (23 cd/m^2^). They had to press a button in response to these luminance changes (with response hand changing from left to right, or vice versa, for each successive block). Luminance changes occurred on 25% of all trials (16 trials per block) simultaneously with the onset of the face array, and remained present for 200 ms until face array offset. These luminance change trials were equally often accompanied by face arrays with neutral or with fearful singletons on the left or right side. In the remaining 48 trials per block, the fixation cross remained dark grey, and no response was required. Twenty-four of these no-change (non-target) trials contained a neutral singleton among fearful faces on the left or right side, and 24 other no-change trials contained a left or right fearful singleton among neutral faces. All different trial types were presented in random order.

### 1.3 Data acquisition and analysis

Scalp potentials were recorded from 23 Ag/AgCl electrodes mounted in an elastic cap according to the International 10–20 System at sites (FPz, F7, F3, Fz, F4, F8, FC5, FC6, T7, C3, Cz, C4, T8, CP5, CP6, P7, P3, Pz, P4, P8, PO7, PO8, Oz), referenced to linked earlobes. The electro-oculogram (EOG) was recorded between electrodes placed at the outer canthus of each eye to monitor horizontal eye movements, and an electrode above the right eye was used to monitor eyeblinks. EEG activity was amplified with a bandpass of DC to 40 Hz, and digitized at a sampling rate of 200 Hz.

EEG was epoched offline into 800 ms intervals, from 100 ms before to 700 ms after stimulus onset. Artifact rejection was performed by removing epochs with activity exceeding ±30 μV in the horizontal EOG channel, ±60 μV in the vertical EOG channel, and ±80 μV for any other electrode. Trials with incorrect responses (missed luminance changes, false alarms on no-change trials) were excluded from analysis. Separate averages were computed for luminance change and no-change trials as a function of singleton type (fearful versus neutral) and singleton location (left versus right). Analyses focused on lateral posterior electrodes PO7 and PO8, where the N2pc component is maximal. The N2pc was quantified on the basis of ERP mean amplitudes measured at PO7 and PO8 within two successive time windows (early N2pc: 170 −220 ms post-stimulus; late N2pc: 225–270 ms post-stimulus).

## 2 Results

### 2.1 Behavioural results

Mean response time to changes in the luminance of the fixation cross (472 ms) was not significantly affected by singleton type or singleton location. Participants missed 2.5% of all luminance changes and responded incorrectly on 0.6% of all trials where no luminance change occurred. Error rates were not significantly affected by singleton type or location.

### 2.2 ERP results

#### 2.2.1 No-change trials

**Fig. 2:**
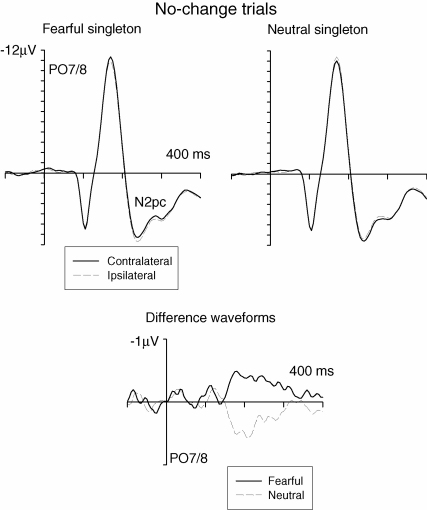
Top: grand-averaged ERPs elicited in no-change trials to arrays containing a fearful singleton face among neutral faces (left panel), or a neutral singleton face among fearful faces (right panel) at electrodes PO7/8 contralateral (solid lines) and ipsilateral (dashed lines) to the singleton. Bottom: difference waveforms obtained by subtracting ERPs at electrodes ipsilateral to the singleton face from contralateral electrodes for trials with fearful (solid line) or neutral (dashed line) singletons.

Fig. 2 (top) shows ERPs obtained at electrodes PO7/PO8 contralateral to the singleton location (solid lines) and ipsilateral to the singleton (dashed lines) on trials where no response-relevant luminance change occurred, for arrays containing a fearful singleton face among neutral faces (left panel) and for arrays containing a neutral singleton among fearful faces (right panel). An enhanced negativity was observed contralateral to the fearful face singleton (N2pc), with its early phase overlapping with the N1 component. An effect of opposite polarity (an ipsilateral negativity) was observed for trials containing a neutral face singleton. This can be seen more clearly in the difference waveforms in Fig. 2 (bottom), which were obtained by subtracting ERPs elicited ipsilaterally to the singleton from ERPs obtained contralaterally. For fearful singletons (solid lines), an enhanced contralateral negativity started about 170 ms post-stimulus, consistent with N2pc components previously observed during visual search (c.f., Woodman and Luck, 1999). A lateralised effect of opposite polarity was obtained for neutral singleton faces (dashed lines).

In repeated-measures analyses of variance (ANOVA) with the factors singleton type, singleton location, and contralaterality (electrode contralateral versus ipsilateral to the side of the singleton), significant singleton type × contralaterality interactions were obtained (*F*(1,15) = 18.9 and 6.9; *p* < .001 and .02, for the early and late N2pc time window), reflecting the fact that lateralised effects of opposite polarity were obtained for trials with fearful and neutral singleton faces. Follow-up analyses conducted separately for these two trial types revealed significant effects of contralaterality for trials with fearful and neutral singletons in both time windows (all *p* < .05) As the ‘reversed’ N2pc for trials with neutral singleton faces is likely to reflect an N2pc that is triggered by the fearful face located next to fixation (see Section 3), additional analyses were conducted across trials with fearful and neutral singletons, with contralaterality now defined relative to the location of the fearful face close to fixation (so that the singleton type × contralaterality interactions observed in the previous analyses now appeared as main effects of contralaterality). No contralaterality × singleton type interactions were found (both *F* < 1), thus confirming that lateralised effects of equivalent size were triggered in response to both types of stimulus arrays (see Fig. 2).

#### 2.2.2 Luminance change trials

**Fig. 3:**
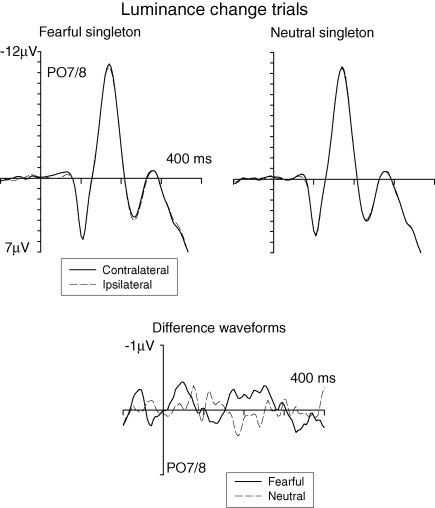
Top: grand-averaged ERPs elicited in luminance change trials containing a fearful singleton face among neutral faces (left panel), or a neutral singleton face among fearful faces (right panel) at electrodes PO7/8 contralateral (solid lines) and ipsilateral (dashed lines) to the singleton. Bottom: difference waveforms obtained by subtracting ERPs at electrodes ipsilateral to the singleton face from contralateral electrodes for trials with fearful (solid line) or neutral (dashed line) singletons.

Fig. 3 shows ERPs elicited at PO7/PO8 contralateral and ipsilateral to the singleton on trials where a target luminance change occurred, for fearful singletons among neutral faces, and neutral singletons among fearful faces (right panel), together with difference waveforms computed by subtracting ipsilateral from contralateral ERPs as a function of singleton location. Although any lateralised effects appear small in these waveforms, statistical analyses revealed the presence of a reliable early N2pc. This was reflected by a main effect of contralaterality (defined relative to the location of the fearful face next to fixation) in the early N2pc window (170–220 ms post-stimulus; *F*(1,15) = 5.3; *p* < .04). In contrast, no significant effect of contralaterality was present for the late N2pc interval (*F* < 2). To explore whether the early N2pc was reduced in luminance change relative to no-change trials, difference waveforms were computed by subtracting ERPs at PO7/PO8 ipsilateral to the fearful face next to fixation from contralateral ERPs, and a planned contrast was used to compare N2pc amplitudes obtained for both types of trials between 170 and 220 ms post-stimulus. A significant difference was obtained (*t*(15) = 1.8; *p* < .05, one-tailed), thus indicating that the presence of a simultaneous task-relevant luminance change at fixation attenuated the N2pc in response to task-irrelevant fearful faces.

## 3 Discussion

An N2pc was elicited in response to task-irrelevant fearful face singletons among neutral faces on no-change trials. This finding demonstrates that fearful faces can bias the spatial distribution of attention even when attention is allocated to a continuous visual monitoring task at fixation, and peripheral faces can be entirely ignored. The fact that an N2pc was still present shows that the attentional capture by emotionally salient events is not restricted to situations when attention is initially unfocussed (as in previous behavioural visual search studies where observers were preparing to find a target in a visual search array; e.g., Öhman et al., 2001a, 2001b), but can also be triggered by task-irrelevant fear-related stimuli that are presented outside a narrow central focus of attention.

The enhanced negativity triggered ipsilateral to a neutral face singleton among fearful faces on no-change trials is most likely to represent an N2pc triggered by the fearful face located next to fixation, and opposite to the neutral face singleton. Given that attention was narrowly focused at the screen centre, and all but the two innermost faces were centred at eccentricities of 8.9° of visual angle and beyond (see Fig. 1), only the face pair closest to fixation may have been processed to the level where emotional expression could be discriminated. If this was the case, arrays containing fearful and neutral singletons would be equivalent in terms of attentional capture by fearful faces, as both contain one fearful and one neutral face close to fixation, and peripheral faces that remain subjectively indeterminate in terms of emotional content. In line with this assumption, the N2pc observed for fearful face singletons and the ‘reversed’ N2pc to neutral face singletons were equivalent in terms of their time course and amplitudes (Fig. 2, bottom).

Finally, N2pc amplitudes were reduced on trials where a task-relevant luminance change occurred at fixation simultaneously with the onset of the face array relative to no-change trials. The attenuation of the N2pc on these trials suggests that the presence of a target, and associated target identification, response selection, and response execution processes, reduce the ability of emotionally salient peripheral stimuli to capture attention. Analogous reductions of N2pc amplitudes as a function of concurrent target processing have recently been reported in ERP studies investigating the attentional blink (Jolicoeur et al., 2006, in press). In these experiments, the N2pc to peripheral targets was attenuated when these were presented in close temporal proximity to a previous task-relevant stimulus, suggesting that the consolidation of target events in visual short-term memory interferes with subsequent shifts of attention. This may also explain why previous studies in our lab (Eimer et al., 2003; Holmes et al., 2003) have found that ERP effects of emotional facial expression processing are eliminated for emotional faces at unattended locations, suggesting that the processing of emotional faces is strongly gated by spatial attention. In these studies, unattended faces were always presented simultaneously with other target stimuli, and the processing of non-face targets may have been sufficient to prevent attentional capture.

In summary, the present results provide new electrophysiological evidence for the hypothesis that task-irrelevant fearful faces can trigger attentional capture even when attention is narrowly focused. Capture is reduced by the simultaneous presentation of a target event. Future experiments need to investigate whether analogous results can also be obtained for other facial expressions. For example, attentional capture may be even more pronounced in response to an immediate threat signaled by angry faces.
